# Brain morphometry and connectivity differs between adolescent‐ and adult‐onset major depressive disorder

**DOI:** 10.1002/da.23254

**Published:** 2022-04-14

**Authors:** Thomas S. Blank, Bernhard M. Meyer, Marie‐Kathrin Wieser, Ulrich Rabl, Paul Schögl, Lukas Pezawas

**Affiliations:** ^1^ Department of Psychiatry and Psychotherapy Medical University of Vienna Wien Austria

**Keywords:** affective disorders, illness onset, major depressive disorder, neuroimaging, psychiatry, voxel‐based morphometry

## Abstract

**Background:**

Early‐onset (EO) major depressive disorder (MDD) patients experience more depressive episodes and an increased risk of relapse. Thus, on a neurobiological level, adult EO patients might display brain structure and function different from adult‐onset (AO) patients.

**Methods:**

A total of 103 patients (66 females) underwent magnetic resonance imaging. Structural measures of gray matter volume (GMV) and functional connectivity networks during resting state were compared between EO (≤19 years) and AO groups. Four residual major depression symptoms, mood, anxiety, insomnia, and somatic symptoms, were correlated with GMV between groups.

**Results:**

We found comparatively increased GMV in the EO group, namely the medial prefrontal and insular cortex, as well as the anterior hippocampus. Functional networks in EO patients showed a comparatively weaker synchronization of the left hippocampus with the adjacent amygdala, and a stronger integration with nodes in the contralateral prefrontal cortex and supramarginal gyrus. Volumetric analysis of depression symptoms associated the caudate nuclei with symptoms of insomnia, and persisting mood symptoms with the right amygdala, while finding no significant clusters for somatic and anxiety symptoms.

**Conclusions:**

The study highlights the important role of the hippocampus and the prefrontal cortex in EO patients as part of emotion‐regulation networks. Results in EO patients demonstrated subcortical volume changes irrespective of sleep and mood symptom recovery, which substantiates adolescence as a pivotal developmental phase for MDD. Longitudinal studies are needed to differentiate neural recovery trajectories while accounting for age of onset.

## 1 INTRODUCTION

Major depressive disorder (MDD) is a debilitating and common disease affecting about 6%–10% of the adult population worldwide each year (Hasin et al., 2018; Malhi and Mann, 2018). The onset of MDD occurs at almost any age throughout the human lifespan (Malhi and Mann, 2018; Otte et al., 2016), but most patients develop their index episode between mid‐adolescence and their early 40s (Kessler and Bromet, 2013). According to the STAR*D study, more than 37% of patients experience their index episode before adulthood (Zisook, Lesser, et al., 2007; Table 1) showing a distinct course of illness compared to adult onset (AO), which is reflected in the diagnostic and statistical manual of mental disorders, fifth edition (DSM‐5) age of onset specifier for depressive disorders (American Psychiatric Association, 2013). These salient differences between adolescent‐ and AO are not only apparent on a clinical, but also on a molecular level, which raises the possibility of distinct pathological entities sharing the same psychopathology (Kaufman et al., 2001).

**Table 1:** Clinical characteristics and residual symptoms

	Early Onset	Adult Onset	*t*/*χ* ^2^
*n* (female)	56 (39)	47 (27)	1.16 (*χ* ^2^)
Age, years	25.77 ± 5.12	27.11 ± 5.01	−1.34
Onset, years	16.23 ± 2.1	23.15 ± 2.98	−13.24***
Education, years	12.46 ± 1.07	12.49 ± 1.6	−0.09
German vocabulary test (WST)	32.81 ± 5.37	33.36 ± 2.53	−0.66
HAMD	1.79 ± 1.89	1.94 ± 1.72	−0.42
Number of past MDEs	2.59 ± 2.67	1.45 ± 1.16	2.9**
Proportion of first‐degree relatives with reported mood disorders	0.55	0.34	3.86* (*χ* ^2^)
Residual mood symptoms	0.029 ± 0.044	0.025 ± 0.04	−0.37
Residual anxiety symptoms	0.031 ± 0.083	0.033 ± 0.066	0.14
Residual insomnia symptoms	0.098 ± 0.161	0.128 ± 0.181	0.86
Residual somatic symptoms	0.039 ± 0.09	0.043 ± 0.101	0.2

Abbreviations: HAMD, Hamilton depression rating scale; MDE, major depressive episode; WST, Wortschatztest, 1992.

* *p* < .05 shows significant *t*‐test or *χ* ^2^ test.

** *p* < .01 shows significant *t*‐test or χ^2^ test.

*** *p* < .001 shows significant *t*‐test or *χ* ^2^ test.

In line with previous studies (Bartova et al., 2015; McGlashan, 1989; Pajer et al., 2012), we define early (adolescent) onset (EO) as the occurrence of the depressive index episode before the age of 20. Compared to AO, EO patients have more familial cases (Kendler et al., 2005) and are more likely to develop substance use or personality disorders (Zisook, Rush, et al., 2007). They also have a higher risk for conversion to bipolar disorder compared to AO patients (Yalin and Young, 2019), which is in line with their genetic profile (Power et al., 2017). EO patients are more prone to develop a recurrent and chronic course of depression (Gollan et al., 2005; Korczak and Goldstein, 2009). As a consequence, EO depression is associated with increased hospitalization (Korczak and Goldstein, 2009), relapse (Gollan et al., 2005), and treatment resistance (Schosser et al., 2012).

Cerebral rewiring and pruning of synapses from adolescence to a person's early 20s is thought to have a lifelong impact on neural efficiency, on how the brain integrates information (Dahl et al., 2018; Foulkes and Blakemore, 2018; Schmaal et al., 2017). Adolescence is a crucial period for emotion regulation and implicated circuitries, particularly so for the affective feedback loop between prefrontal and subcortical regions (Mayberg, 1997; Roiser et al., 2012). During adolescence, an increasing functional integration of subcortical regions and the prefrontal cortex (PFC; Dahl et al., 2018; Straub et al., 2019) drives maturation of higher‐order cognitio (Andersen and Teicher, 2008; Calabro et al., 2020).

A large body of imaging studies testifies to the specific burden EO‐MDD patients bear including patients at risk (Little et al., 2014; Swartz et al., 2015; Whittle et al., 2011), in an early major depressive episode (MDE, Geng et al., 2016; Ho et al., 2017) (Redlich et al., 2018), or after remission (Barch, Harms, et al., 2019; Jacobs et al., 2014). Combined with no less than 34 independent, longitudinal pediatric samples, these studies highlight the prominent role of the hippocampus and the amygdala (Toenders et al., 2019) as part of emotion regulation networks in EO depression. One study between adult EO‐ versus AO‐MDD patients indicated alterations in resting state functional connectivities (RSFCs) between the amygdala and cortical regions (Clark et al., 2018). Another yielded less conclusive structural results (Jaworska et al., 2014). However, none of them controlled for illness severity in a sufficiently powered functional and structural magnetic resonance imaging (MRI) study. Extending our previous study, we compare EO versus AO in remitted major depressive disorder (rMDD) patients to attenuate the effect of acute symptom load and thus, presumably, physiological variance.

Several routes are plausible through which deficient brain functioning exposes individuals to a heightened MDD susceptibility during adolescence. On the one hand, a difference in brain morphometry and functioning is expected in adult EO patients, since they demonstrate heightened vulnerability to genetic and environmental determinants even before their index MDE (Kendler and Gardner, 2016). Emotional responsiveness and sensitivity to environmental stressors during adolescence may act as MDD triggers (Belsky et al., 2009; Homberg, 2012; Vinogradov et al., 2012). Dysfunctional emotion regulation, a process subsequent to an emotional response, is a well‐known feature of MDD corresponding to the interplay of amygdala and hippocampus (Phillips et al., 2015; Vanderlind et al., 2020).

On the other hand, the concept of stress‐induced neurobiological “scars,” kindling or sensitization, respectively, has often been invoked to explain the recurrent nature of MDD (Bos et al., 2018; Post, 2007). Particularly for the hippocampus, the well‐researched curtailment of neurogenesis by stress‐induced glucocorticoid secretion is a candidate mechanism for such a neurobiological scar (Andersen and Teicher, 2008). For EO patients, we could hypothesize that heightened vulnerability during critical developmental phases could lead to an increased likelihood for recurrent episodes. It has been suggested that MDD vulnerability might occur during periods of very rapid development, which might unmask underlying predispositions (Andersen and Teicher, 2008; Lupien et al., 2009).

Our first aim was to assess structural and functional differences between EO and AO patients. As outlined above, MDD neuroimaging results often appear equivocal due to varying symptom loads (American Psychiatric Association, 2013). By recruiting rMDD patients, we hoped to limit heterogeneity of severity during different stages of the illness course (Meyer et al., 2019). Accordingly, we compared EO‐ versus AO‐rMDD patients using morphometry and RSFC. We hypothesized that EO patients compared to AO patients would exhibit altered volumes and RSFC in the PFC (Schmaal et al., 2016) and subcortical regions associated with emotion regulation (Clark et al., 2018; Roiser et al., 2012; Whittle et al., 2014). To add a layer of clinical explanation to anatomical and functional findings, the paper's second aim was to analyze the interaction of age of onset and the presence of four MDD symptom dimensions with gray matter volume (GMV).

## 2 MATERIALS AND METHODS

### 2.1 Subjects

This neuroimaging study was carried out at the Department of Psychiatry and Psychotherapy of the Medical University of Vienna (MUV), Vienna, Austria, in cooperation with the Department of Psychology, Dresden University of Technology, Dresden, Germany. rMDD patients enrolled via public advertising and consented in writing. One fully remitted MDE was required for inclusion. Subjects with previous or current axis I disorder were excluded. To increase the validity and clinical significance of retrospectively assessed anamnestic information, only patients who had received pharmacological or psychotherapeutic treatment were included. Furthermore, no psychopharmacological drug treatment was allowed for at least 3 months before recruitment. The study protocol was approved by the Ethics Committee of the MUV (Ethics Committee Number: 11/2008) under the Declaration of Helsinki (World Medical Association, 2013). A detailed description of recruitment procedures, exclusion/inclusion criteria, and clinical assessments can be found in previous work (Bartova et al. 2015). Briefly, the assessment included psychiatric examination establishing diagnosis via the German version of the Structured Clinical Interview for DSM‐IV Axis I disorders (Wittchen et al., 1997). The severity of depression symptoms was assessed through the 17‐item Hamilton depression rating scale (HAMD; Hamilton, 1986). A 17‐item HAMD score of ≤5 (Romera et al., 2011) was required for inclusion, which ensured that MDD patients met criteria for a current rMDD status. Subjects underwent MRI scans with structural and resting state imaging sequences.

### 2.2 Scanner parameters

MRI data were acquired on a 3 Tesla (3 T) TIM Trio scanner equipped with a Siemens 12‐channel head coil at both research sites. The same protocol for scanning and quality controls was established at both sites (Rabl et al., 2014). During and after acquisition, visual inspection was carried out to ascertain good quality of data. Through the use of foam pads and movement parameters, head movements were both minimized and quantified.

Resting state MRI data were acquired via a phase‐corrected blipped gradient echo, single‐shot echo‐planar imaging sequence (echo time [TE]/repetition time [TR] = 42/2000 ms, 96 × 96 matrix, 210 mm square field of view [FoV], 20 axial slices, slice thickness = 4 mm, slice gap = 1 mm) employing an interleaved slice acquisition scheme. Structural MRI data were acquired with a three‐dimensional MPRAGE sequence (TR/TE = 2300/4.21 ms, 240 × 256 × 176 mm FOV, flip angle 9 degrees, inversion time 900 ms, voxel size of 1 × 1 × 1.1 mm) during the same session as the functional MRI (fMRI) data.

### 2.3 Preprocessing

Structural T1‐weighted images were preprocessed with the computational anatomy toolbox (CAT12) for SPM (Gaser and Dahnke, 2016). Preprocessing steps consisted of skull stripping, gray and white matter segmentation, and normalizing to a 1.5 mm structural Montreal Neurological Institute (MNI) template. Subsequently, spatial Gaussian kernel smoothing of 10 mm full width at half maximum (FWHM) was applied. Gray matter segmentation in CAT12 was used to approximate the total intracranial volume of subjects.

Functional data were preprocessed utilizing the CONN toolbox's standard preprocessing pipeline (Nieto‐Castanon, 2020). Succinctly, steps included slice‐time correction, MNI normalization, and blurring at 10 mm FWHM. Linear regression of nuisance signals included an anatomical (white matter and cerebrospinal area) component‐based noise correction procedure (aCompCor), 12 subject‐motion parameters and scrubbing of identified outlier volumes (Nieto‐Castanon, 2020). Moreover, blood oxygenation level‐dependent time series were bandpass filtered to frequencies between 0.008 and 0.09 Hz.

### 2.4 Statistics

The main variable of interest, depression onset, was derived via binarizing patients’ reported age of their index MDE using a cutoff of ≤19 years for EO and >19 years for AO (Bartova et al., 2015; McGlashan, 1989; Pajer et al., 2012). The symptom dimensions, insomnia, mood, anxiety, and somatic symptoms were derived by adopting Shafer (2006) factor analysis of the HAMD. Symptom dimensions were then calculated as the means of the subsets of single HAMD items pertaining to Shafer (2006) several factors. Symptom dimensions were further binarized, that is, symptom_binary_ = {0 if symptom = 0; 1 else} to exclude a potential leverage effect of patients with multiple symptoms in one factor. Hence, we increased the analysis’ sensitivity for a weak symptom load in each factor taking into account that a HAMD sum score of five was mandatory for subject inclusion.

SPM was used to compute group‐level correlations of GMV from first‐level segmentations. First, an analysis of covariance (ANCOVA) model was computed to uncover whole‐brain volumetric differences between EO and AO patients. For this principal analysis, the cluster forming threshold was set to *p*_uncorrected_ = .0025 (Takeuchi et al., 2012). For multiple comparison corrections, we set the Bonferroni‐corrected family‐wise error (FWE) threshold to *p*_FWE_ < .01 to allow correction for the five ANCOVA models, including the subsequently described four models.

For subanalyses of HAMD factors, we computed the same model replacing the variable of interest with interactions of onset × insomnia, onset × mood, onset × anxiety, and onset × somatic symptoms, respectively. Subanalyses were computed within a dilated depression mask derived from neurosynth.org (Yarkoni et al., 2011; details in Figure S2) using a cluster forming threshold of *p*_uncorrected_ = .01. Age, gender, and total intracranial volume were used as nuisance covariates in all anatomical models. Post hoc *t*‐tests were calculated for the four interaction models’ largest, significant clusters. *p*‐values of these were Bonferroni‐corrected to account for six between‐group tests per interaction effect.

The CONN toolbox’ RSFC network analysis was used to test functional interrelations of depression related and most significant regions (Table S3) found in the voxel‐based morphometry analysis mediated by age of onset. To ensure standardization of brain regions, the Harvard–Oxford atlas as implemented in CONN was used to define anatomical boundaries for the network nodes. An ANCOVA model with onset as the variable of interest using age and gender as nuisance variables was computed. The network connectivity statistics were corrected for multiple testing via *p*_FDR_ < .05. *Z*‐values of significant nodes in the network were extracted, plotted, and retested individually to corroborate the network connectivity's result. Further details regarding CONN network statistics and ROI selection are available in the Supplement.

## 3 RESULTS

### 3.1 Clinical characteristics

A total of 103 patients (66 females, age mean = 26.38 ± 5.1) had anatomical scans available (Table 1). For resting state analysis, a subsample of 64 patients was available.

Table 1 shows that the two onset groups differ significantly in clinical factors: age of onset, number of past MDEs, and proportion of first‐degree relatives with mood disorders. No significant difference was observed for demographic variables gender proportion, age, education, and vocabulary test. Neither was there any significant difference in symptom severity, that is, HAMD score or residual symptom dimensions.

### 3.2 Morphometric analysis

**Figure 1:**
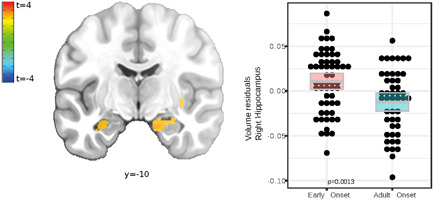
Coronal slice (*p*_uncorrected_ = .0025) and scatter plot display right anterior hippocampus volumes differing between onset groups. Colored boxes show the mean and bootstrapped 95% confidence intervals. Post hoc *t*‐test test for right anterior hippocampus gray matter volume residuals corrected for age, gender, and total intracranial volume, was significant between groups (*t* = 3.28, *df* = 102, *p* < .01).

The principal anatomical analysis was conducted to reveal brain structural differences between EO and AO of rMDD patients. Significant clusters, including depression relevant regions, such as the bilateral amygdala (Figure 1), anterior cingulate, and the insular cortex, exhibited higher volume in EO patients (Table 2). Post hoc models (Table S1) explained more variance with age of onset as predictor compared to alternative, highly correlative, clinical variables such as number of depressive episodes (nDE) and time in depressive episodes.

**Table 2:** Significant clusters of anatomical analyses for the onset main model and two models analyzing interaction effects onset × mood and onset × insomnia (*p*_uncorrected_ = .01, *p*_FWE_ < .01), respectively

Region	Voxels in cluster	*t* cluster mean	*t* peak	Partial e2	*df*	*x*, *y, z *peak (MNI; LPI)
**Main model: Onset**						
Left anterior hippocampus	119	3.02	3.53	0.3	98	−34 −12 −18
Left superior temporal gyrus	325	3.14	3.55	0.28	98	−63 −33 13
Right ventromedial prefrontal cortex	194	3.07	3.48	0.41	98	9 45 −8
Right anterior hippocampus	164	3.02	3.44	0.39	98	15 −10 −22
Right insular cortex	133	3.09	3.47	0.38	98	36 13 −16
Right posterior insula	190	3.05	3.37	0.42	98	36 −8 −3
Right superior frontal gyrus	145	3.12	3.55	0.26	98	21 57 4
**Interaction model: Onset × insomnia**						
Left caudate	98	2.48	3.33	0.07	96	15 −4 9
Right caudate	100	2.52	3.21	0.08	96	−11 −3 19
**Interaction model: Onset × mood**						
Right amygdala	271	2.5	2.74	0.07	96	34 −1 −22

Abbreviations: *df*, degrees of freedom; FWE, family‐wise error; LPI, orientation left posterior–inferior; MNI, Montreal Neurological Institute.

**Figure 2:**
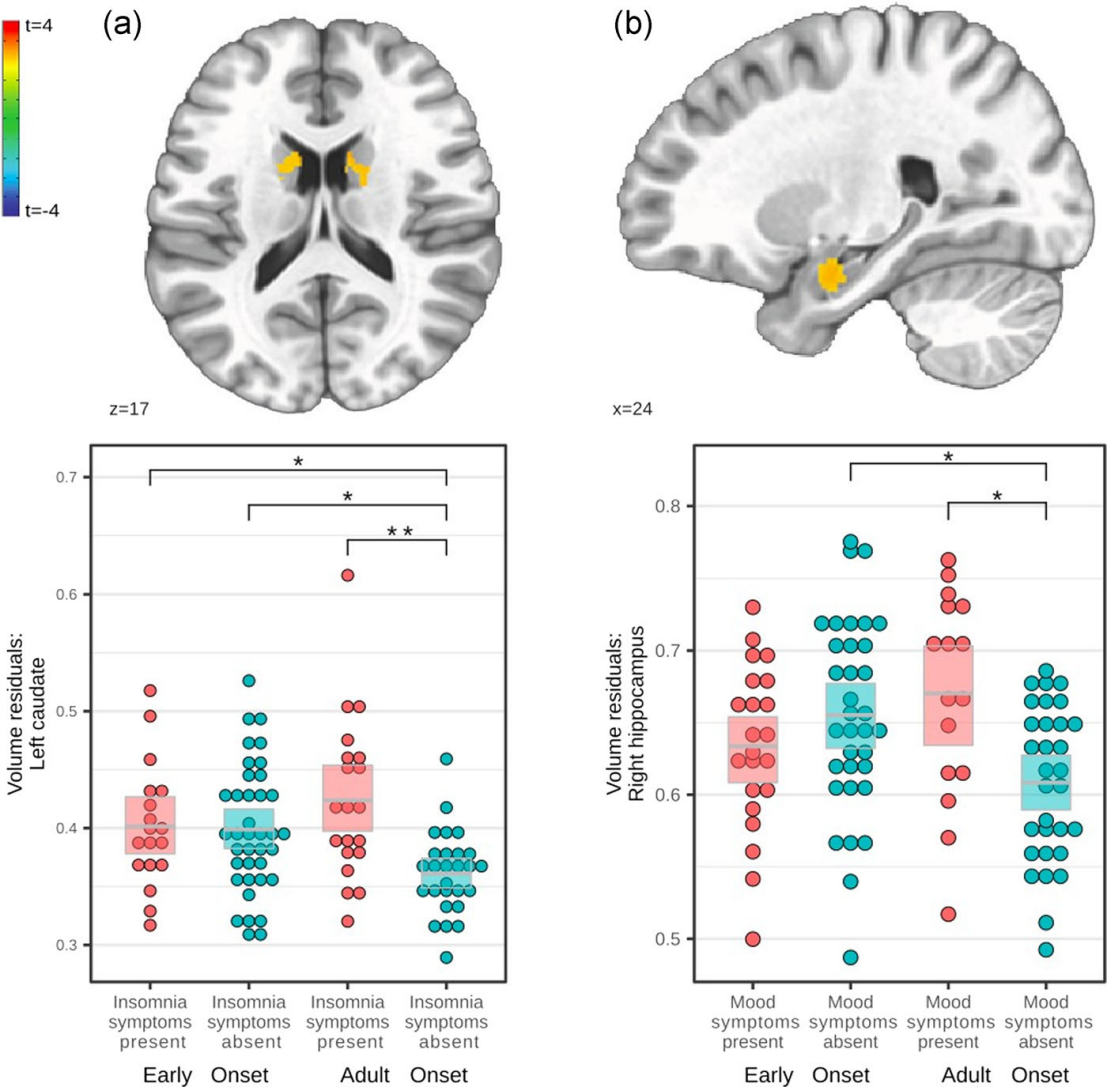
(a) Slices (top) and scatter plots (bottom) showing left caudate volumes (left) and (b) right hippocampus volumes (right) correlating with the interaction term onset × insomnia (left) and onset × mood (right), respectively. Slices are shown at *p*_uncorrected_ = .01. Volume residuals were derived from gray matter volume by correcting for age, gender, and total intracranial volume. *p < .05 significant *t*‐test, **p < .01 significant *t*‐test.

Significant clusters for all four HAMD factors were found, but post hoc between‐group tests were nonsignificant for all anxiety and somatic symptom clusters (Figure S1 and Table S2). Significant clusters for insomnia, bilateral caudate nucleus, and mood symptoms, right amygdala, are shown in Figure 2 and Table 2, including significant post hoc between‐group tests.

### 3.3 Resting state network analysis

**Figure 3:**
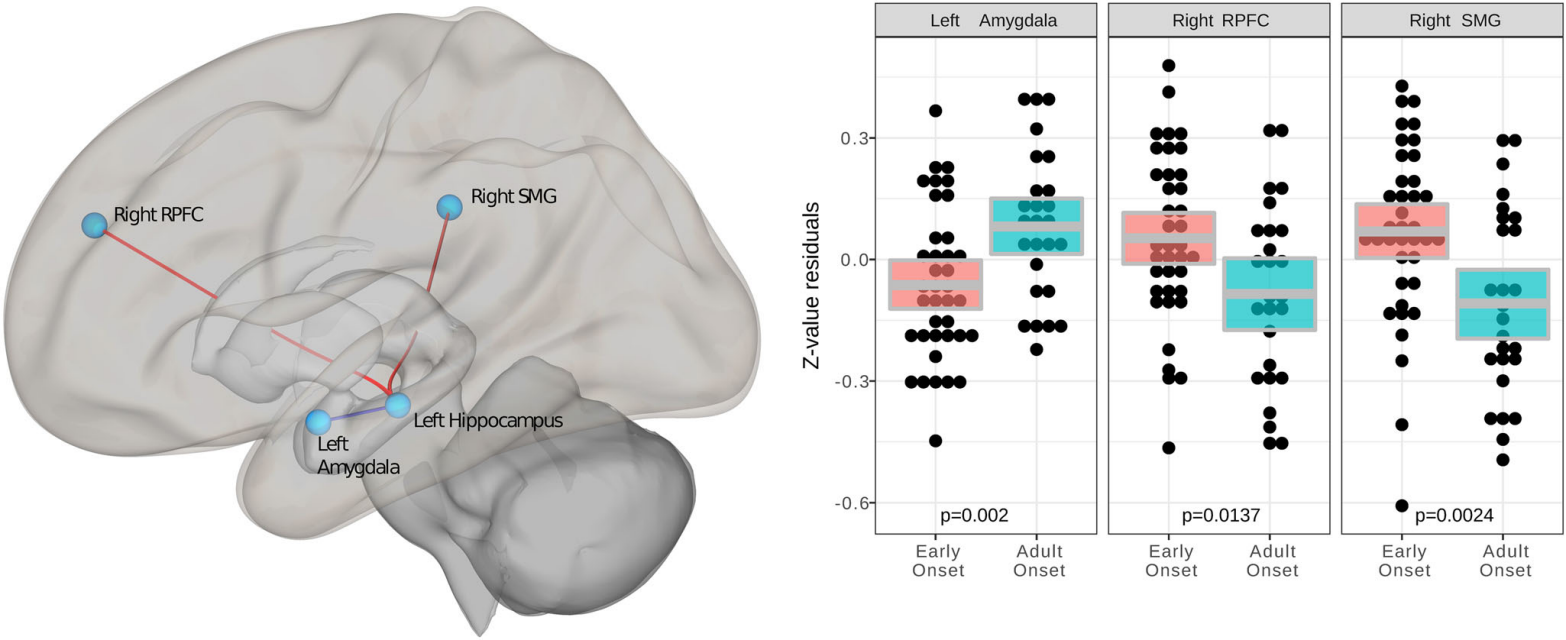
(Left) Three‐dimensional brain model of RSFC network analysis. The left hippocampus is the central hub in this network. (Right) The scatter plots show RSFC residuals between the left hippocampus and peak regions with strongest age of onset effects corrected accounting for age and gender. Colored boxes show the mean and bootstrapped 95% confidence intervals, *p*‐values are displayed at the bottom of the graphs, respectively. CONN, RSFC toolbox; RPFC, rostral prefrontal cortex; RSFC, resting state functional connectivity; SMG, supramarginal gyrus

In the second analysis, we used resting state MRI data to facilitate interpretation and complement structural findings on a network level (Figure 3).

When comparing EO versus AO, the network's main hub was the left hippocampus (*F*(13, 48) = 1.94, *p*_FDR_ = .048). Post hoc tests of connectivity between the left hippocampus and other regions of interest (Figure 3) for EO subjects resulted in a) weaker connectivity with the ipsilateral amygdala (*t*(60) = −3.18, *p*_FDR_ = .042), and b) an oppositional, stronger RSFC with the contralateral PFC (*t*(60) = 2.75, *p*_FDR_ = .047) and supramarginal gyrus (SMG) (*t*(60) = 2.75, *p*_FDR_ = .047) as compared to AO subjects.

## 4 DISCUSSION

Our structural and functional findings demonstrate that age of onset is related to alterations in emotion‐regulation networks (Mayberg, 1997; Pessoa, 2008; Roiser et al., 2012) even in rMDD patients with minor residual symptoms. We found increased GMV in the ventromedial PFC (vmPFC), the insula, as well as the hippocampus. Complementing these structural findings, RSFC showed a weaker synchronization between the hippocampus and the amygdala in EO patients, but stronger connectivity between the hippocampus and contralateral PFC as well as SMG. Hence, the importance of the hippocampus and PFC regarding age of onset was not only exhibited morphologically but corroborated functionally. Finally, analysis of the four residual symptom dimensions via the HAMD revealed reduced volumetric sensitivity in adolescent‐onset patients; in the hippocampus for mood symptoms, and in the caudate nuclei for insomnia, and no significant clusters for somatic and anxiety symptoms.

In all analyses, the central brain region was the hippocampus (Figures 1, 2, 3). Hippocampus volume alterations are frequently reported in acute MDE (Wise et al., 2017; Zavorotnyy et al., 2018) at early subclinical stages of depression (Barch, Tillman, et al., 2019; Henje Blom et al., 2015; Redlich et al., 2018) and as a predictor of depression even years before onset (Carballedo et al., 2012; Whittle et al., 2011). The vast number of developmental findings in children and adolescence underline the importance of the hippocampus for EO depression.

Higher hippocampus volume in EO patients (Figure 1, Table 2) might reflect maladaptive emotion regulation due to both environmental stress and genetic vulnerability during development (Kendler et al., 2001; Teicher et al., 2016). Loss of hippocampus volume was previously shown in symptomatic (Barch, Harms, et al., 2019; Henje Blom et al., 2015) and in asymptomatic children affected by environmental risk factors like childhood maltreatment (Redlich et al., 2018), maternal aggression (Whittle et al., 2011), and emotional abuse (Carballedo et al., 2012). MDE prediction studies using hippocampal volume might indicate the region's mediating role between environmental stress and an early illness onset (Carballedo et al., 2012; Little et al., 2014). Specifically, a larger left hippocampus, as observed in EO patients (Figure 1), was previously identified as a predictor of future depressive episodes for those with past adverse experience (Whittle et al., 2011). According to Whittle et al. (2011), the larger hippocampus might point to an increased environmental sensitivity.

The hippocampal subiculum, the peak GMV alteration locus (Figure 1), plays a central role in emotional downregulation during the experience of psychogenic stressors. In contrast to physical factors, these stressors are based on autobiographical or innate programs (Ulrich‐Lai and Herman, 2009) involved in depressed patients’ memory and perception biases (Young et al., 2013). The subiculum acts as a relay between stress‐inhibitory cortical regions, for example, SMG and rostral PFC, and stress‐excitatory nuclei in the adjacent amygdala (Figure 3 network; Ulrich‐Lai and Herman, 2009).

We observed a remarkable overlap between two imaging modalities: local volume increase in the hippocampus (Figure 1, Table 2), as well as alternated hippocampal functional connectivity in EO patients (Figure 3). The left hippocampus of EO patients showed a weaker RSFC with the adjacent amygdala (Figure 3), and a stronger RSFC with cortical regions including the rostral PFC (Figure 3). This reduced connectivity within limbic regions was previously observed in early‐childhood‐onset depression and also in the asymptomatic family risk subsample (Luking et al., 2011). Luking et al. (2011) explained the lower amygdala‐hippocampus RSFC, as observed here in EO patients (Figure 3), by a malfunctioning integration of emotional experiences in memory and suboptimal coping with sadness. Findings of hippocampus and PFC integration point to dysfunctional top‐down emotion regulation at early stages of depression during adolescence (Geng et al., 2016). Summarizing, the PFC, amygdala and hippocampus coordinate their activity and likely share pathological effects related to an early age of onset in an extensively interconnected emotion‐regulation network (Dahl et al., 2018; Pessoa, 2008; Straub et al., 2019).

Our finding of comparatively increased GMV in the vmPFC (Table 2) mirrors a result reporting heightened volume in EO‐MDD patients in the PFC (Straub et al., 2019). In contrast to subcortical developmental effects, cortical pathogenesis in MDD relies on the idea of a delay in brain maturation (Davey et al., 2008; Straub et al., 2019). Accordingly, one explanation for an increased volume in the vmPFC in EO patients (Table 2) could be a delay in dendritic pruning in adolescence that preserves higher GMV in prefrontal areas than patients with later onset. Still, it remains unclear whether delayed cortical development predisposes a person to EO‐MDD, or whether MDD delays brain maturation (Straub et al., 2019). Early pathology in the emotion‐regulation network comprising the PFC, the hippocampus, and the amygdala might be unexpressed until the PFC would normally subsume affected cognitive control over emotions (Andersen and Teicher, 2008).

As regards persisting symptom dimensions (Figure 2), EO patients showed a dissociation between mood symptoms and hippocampal volumes, as well as between sleep symptoms and bilateral caudate nucleus. Thus, EO patients lacked volumetric sensitivity to the presence or absence of depressive symptoms. Prominent symptoms in adolescent MDD patients are high symptom fluctuation, irritability, and mood reactivity (Kim et al., 2019). In the absence of residual mood symptoms (Figure 2), only AO patients had smaller hippocampi. Further research is needed to understand whether this detachment of volumetric adaption from symptom change is a generalizable pathomechanism. Caudate volume alterations were previously associated with wake‐sleep cycle disruption in rats (Qiu et al., 2010; Vetrivelan et al., 2010) and alterations in total sleep time in humans (Won et al., 2019). Functionally, differences in caudate recruitment in patients with insomnia were exhibited via fMRI during executive tasks (Stoffers et al., 2014) and resting state (Lee et al., 2018). Conjointly, subcortical volumetric sensitivity is less prevalent in EO patients concerning sleep and mood symptoms, for which adolescence is a key developmental phase (Casement et al., 2016).

### 4.1 Strengths and limitations

Although our study employs a sizable rMDD sample from two centers, RSFC datasets were only available from one center. This prohibited testing the four HAMD symptom dimensions between groups via RSFC due to statistical overfitting. An rMDD sample might be more suited to detect developmental neurobiological deviations that would exhibit trait‐like stability, which could be masked by a higher and more acute symptom load. On the other hand, the consequent low symptom heterogeneity deprived our HAMD factors approach of variance, which compelled us to binarize the symptom dimensions' nominal scale.

According to the literature, it seems unlikely that the difference in hippocampus size across the two onset groups was due to the significantly greater number of experienced MDEs in the EO group, since, on the contrary, a higher number of MDEs is associated with a hippocampal volume decline (Hansen et al., 2021). Post hoc analyses clearly showed favorable statistical properties of models with the EO variable compared to alternatives centering on multiple MDE and especially longer duration in MDE (see Table S1, for details). Arguably, if the variables MDD onset and the nDE are high in mutual information, then MDD onset constitutes a better‐suited concept for clinical research. The number and duration of MDE are prone to changes and associated lead‐time and label‐shift biases, but EO is immutable.

## 5 CONCLUSION

This is the first study to show persistent anatomical and functional brain differences in fully remitted MDD patients comparing adolescent to adult age of onset. The convergence of regional morphometry in the hippocampus and its functional integration procure complementary evidence relating the onset of depression to the state of full remission. Not only are these regions robustly implicated in the neurobiology of MDD, particularly in emotion regulation (Pessoa, 2008; Roiser et al., 2012), but they also undergo critical transitions during human brain maturation in tandem (Andersen and Teicher, 2008; Dahl et al., 2018). Interestingly, AO patients showed volumetric differences depending on residual mood and sleep symptoms, respectively, while EO patients demonstrated different regional brain volumes irrespective of symptom recovery. Our results, combined with the existing clinical literature, contribute to the notion that EO‐MDD is an idiosyncratic (sub‐)type of MDD that justifies dedicated research efforts. Future studies are needed to further disentangle MDD's etiology mediated by specific clinical phenomenology during the development of particular brain regions and networks in EO‐MDD.

## AUTHOR CONTRIBUTIONS

Thomas S. Blank, Bernhard M. Meyer, Lukas Pezawas, and Ulrich Rabl designed research. Thomas S. Blank and Bernhard M. Meyer performed research (acquisition, analysis, and interpretation of data). Thomas S. Blank and Bernhard M. Meyer wrote the paper. Lukas Pezawas, Ulrich Rabl, Paul Schögl, and Marie‐Kathrin Wieser critically revised the paper. Lukas Pezawas had full access to all of the data in the study and takes responsibility for the integrity of the data and the accuracy of the data analysis.

## CONFLICTS OF INTEREST

The authors declare no conflicts of interest.

### PEER REVIEW

The peer review history for this article is available at https://publons.com/publon/10.1002/da.23254

## Supporting information

Supplementary information.

## Data Availability

The data that support the findings of this study are available from the corresponding author, Lukas Pezawas, upon reasonable request. The data are not publicly available due to privacy or ethical restrictions.
