# Supplementary material for: Brain morphometry and connectivity differs between adolescent‐ and adult‐onset major depressive disorder
Source: Depress Anxiety. 2022 Apr 14;39(5):387–96. doi: 10.1002/da.23254 (PMC9323432; doi:10.1002/da.23254)
Supplement: Supplementary file 1 — Supplementary information. [file DA-39-387-s001.docx]

Supplement to

# Brain morphometry and connectivity differs between adolescent and adult onset major depressive disorder

## VBM interaction analysis - all symptoms
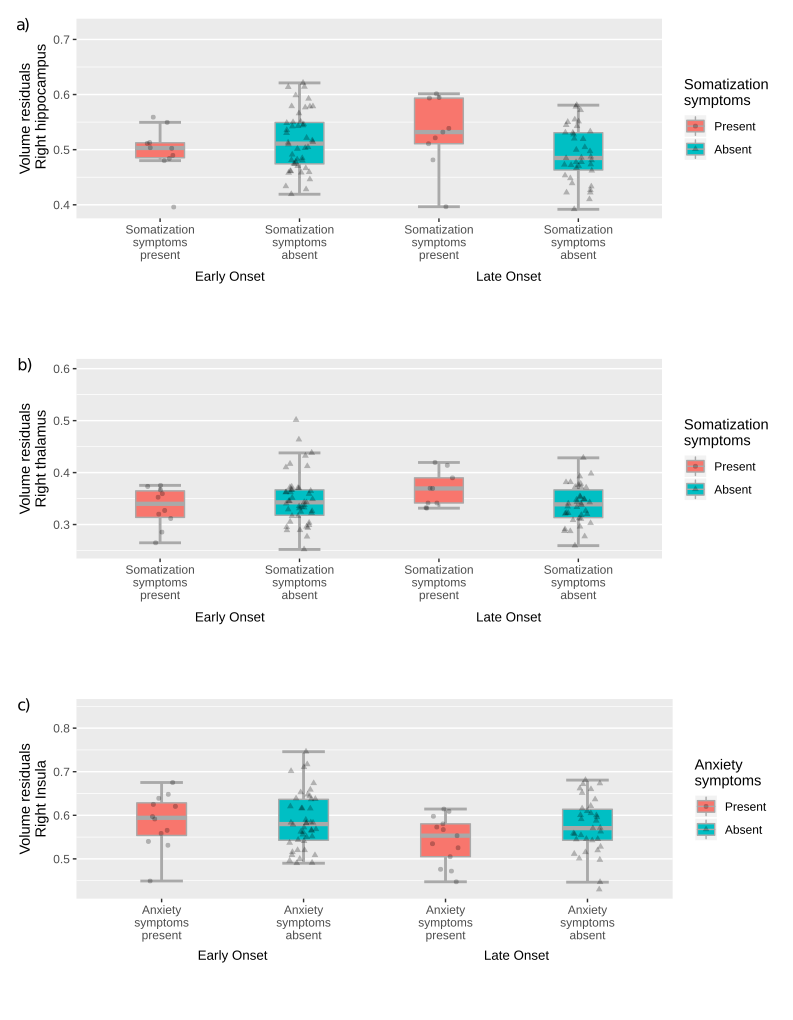


Figure S1. Scatter plots showing groups of the interaction term onset x somatic symptom (a-b) and onset x anxiety (c), respectively. No significant *post-hoc* between-group tests were observed in any of the models.

**Dependent variables:**

| Age  p-value | Sex  p-value | Early Onset  p-value | Multiple nDE  p-value | >median tDE p-value | Adj R2 Full model | AIC |
| --- | --- | --- | --- | --- | --- | --- |
| **Dependent variable: Right Hippocampus Grey Matter Volume (extracted values)** | | | | | | |
| **0.056** | **4.18E-12** | **0.0011** |  |  | **0.54** | **-229.6** |
|  | **1.56E-11** | **0.0039** |  |  | **0.52** | **-227.6** |
| 0.26 | 3.46E-10 |  |  |  | 0.46 | -220.1 |
| 0.56 |  | 0.24 |  |  | 0.008 | -179.9 |
|  | **1.12E-11** | **0.022** | **0.23** |  | **0.52** | **-227.2** |
|  | **1.34E-11** | **0.0024** |  | **0.30** | **0.52** | **-226.8** |
|  | 7.22E-11 |  | 0.034 |  | 0.49 | -223.5 |
|  | 6.05E-10 |  |  | 0.79 | 0.29 | -218.9 |
| **Dependent variable: Left Hippocampus Grey Matter Volume (extracted values)** | | | | | | |
| **0.33** | **3.24E-08** | **0.00065** |  |  | **0.41** | **-201.8** |
|  | **4.24E-08** | **0.00096** |  |  | **0.41** | **-201.8** |
| 0.86 | 2.1E-06 |  |  |  | 0.28 | -190.3 |
| 0.89 |  | 0.067 |  |  | 0.02 | -170.1 |
|  | **8.07E-08** | **0.0012** | **0.58** |  | **0.40** | **-200.2** |
|  | **5.38E-08** | **0.0011** |  | **0.75** | **0.40** | **-199.9** |
|  | 1.62E-06 |  | 0.47 |  | 0.29 | -190.9 |
|  | 2.28E-06 |  |  | 0.62 | 0.29 | -190.6 |

Table S1. Adding Age as a nuisance variable to VBM models (row 1 vs. 2) showed similar prediction for extracted values of the left hippocampus and only slightly better prediction for the right hippocampus (see adjusted R2 and AIC). Replacing AoO by highly correlative clinical variables nDE or tDE lead to a dramatic drop of all displayed model parameters. In other words, models with AoO and Sex (bold rows) outperformed all putative alternatives incorporting both less or not statistically significant clinical variables nDE and especially tDE. Please note that we dichotomized nDE (single vs. multiple episodes) and tDE (median split) due to skewed distributions and to mirror AoO dichotomization. Moreover, AIC of a superior model should be at least 2 points lower than competitors. Abbreviations: AIC, Akaike information criterion; AoO, Age of Onset (AoO); nDE, number of depressive episodes; tDE, time in depressive episodes.; VBM, voxel based morphometry

| Model | Region | Cluster size | t cluster mean | t peak | Partial eta2 | df | x y z peak (MNI; LPI) |
| --- | --- | --- | --- | --- | --- | --- | --- |
| Onset | Left hippocampus | 119 | 3.02 | 3.53 | 0.3 | 98 | -34 -12 -18 |
| Onset | Left superior temporal gyrus | 325 | 3.14 | 3.55 | 0.28 | 98 | -63 -33 13 |
| Onset | Right anterior cingulate cortex | 194 | 3.07 | 3.48 | 0.41 | 98 | 9 45 -8 |
| Onset | Right hippocampus | 164 | 3.02 | 3.44 | 0.39 | 98 | 15 -10 -22 |
| Onset | Right insular lobe | 133 | 3.09 | 3.47 | 0.38 | 98 | 36 13 -16 |
| Onset | Right posterior insula | 190 | 3.05 | 3.37 | 0.42 | 98 | -36 -8 -3 |
| Onset | Right superior frontal gyrus | 145 | 3.12 | 3.55 | 0.26 | 98 | 21 57 4 |
| Onset x Insomnia | Left caudate | 98 | 2.48 | 3.33 | 0.07 | 96 | 15 -4 9 |
| Onset x Insomnia | Right caudate | 100 | 2.52 | 3.21 | 0.08 | 96 | -7 -6 13 |
| Onset x Mood | Right hippocampus | 271 | 2.5 | 2.74 | 0.07 | 96 | -34 1 -22 |
| Onset x Anxiety | Right Insula | 217 | 2.54 | 3.42 | 0.09 | 96 | 45 1 -4 |
| Onset x Somatic symptoms | Right hippocampus | 339 | 2.77 | 3.38 | 0.09 | 96 | 16 -12 -13 |
| Onset x Somatic symptoms | Right thalamus | 91 | 2.57 | 3.49 | 0.07 | 96 | 16 -12 1 |

Table S2. Significant clusters (p_uncorrected_=.0025) of anatomical analyses for the onset main model and four interaction models analyzing the effects onset x insomnia, onset x mood, onset x Anxiety and onset x somatic symptoms (p_uncorrected_=.01). Abbreviations: df, degrees of freedom; LPI, orientation left-posterior-inferior; MNI, Montreal Neurological Institute

## Depression mask specifics for VBM age of onset x symptoms interaction analysis

The depression mask was retrieved from Neurosynth.org [(1)](http://sciwheel.com/work/citation?ids=237365&pre=&suf=&sa=0) on 30.08.2018 using the meta-analytic search term “depression” downloading the “uniformity test” layer (<https://neurosynth.org/analyses/terms/depression/>). According to neurosynth FAQs: “**uniformity test map**: z-scores from a one-way ANOVA testing whether the proportion of studies that report activation at a given voxel differs from the rate that would be expected if activations were uniformly distributed throughout gray matter.” (<https://neurosynth.org/faq/#q18>). The AFNI [(2)](http://sciwheel.com/work/citation?ids=4255219&pre=&suf=&sa=0) neuroimaging software’s command “3dmask_tool” was used to dilate and erode the downloaded map by 5 voxels, respectively, in order to arrive at a more connected, expansive depression mask without holes.


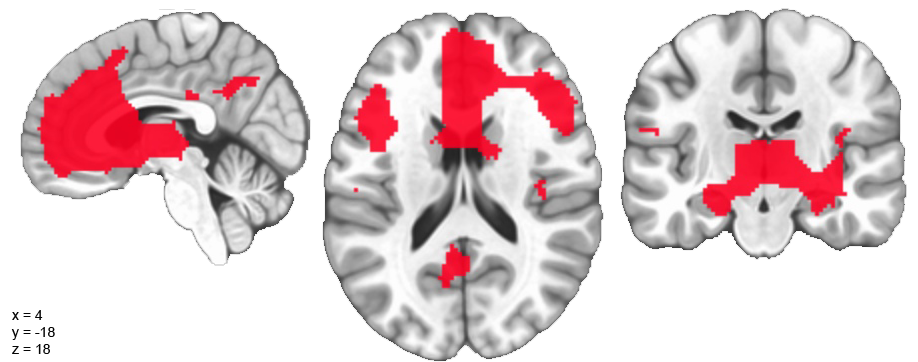


Figure S2. Shows axial, sagittal and coronal slices of the depression mask derived from neurosynth at (x, y, z)_LPI_ = (4, -18, 18).

## ROI-to-ROI RSFC specifics

Resting state networks comprised ROIs of the default network, salience network, fronto parietal network (as defined by the CONN toolbox) and depression relevant subcortical regions (Table S3). Multivariate parametric General Linear Model analyses were conducted on an ROI-level (F-statistics) and for each individual ROI pair (t-statistics) while incorporating age of depression onset and nuisance variables age and gender. F-statistic results for each ROIs intensity and t-statistics for individual connections were FDR-corrected, p<0.05, (Benjamini and Hochberg) for multiple testing.

| **Region** | **Network** | **Coordinates (LPI)** | **CONN internal ROI name** |
| --- | --- | --- | --- |
| Hippocampus (R) | Subcortical ROI | (26, -21, -14) | atlas.Hippocampus r |
| Hippocampus (L) | Subcortical ROI | (-26, -21, -14) | atlas.Hippocampus l |
| Amygdala (R) | Subcortical ROI | (23, -4, -18) | atlas.Amygdala r |
| Amygdala (L) | Subcortical ROI | (-23, -4, -18) | atlas.Amygdala l |
| Posterior cingulate cortex | Default Mode | (1,-61,38) | network.DefaultMode.PCC |
| Medial prefrontal cortex | Default Mode | (1,55,-3) | network.DefaultMode.MPFC |
| Lateral parietal lobe (L) | Default Mode | (-39,-77,33) | network.DefaultMode.LP (L) |
| Lateral parietal lobe (R) | Default Mode | (47,-67,29) | network.DefaultMode.LP (R) |
| Anterior cingulate cortex | Salience Network | (0,22,35) | network.Salience.ACC |
| Anterior insula (L) | Salience Network | (-44,13,1) | network.Salience.AInsula (L) |
| Anterior insula (R) | Salience Network | (47,14,0) | network.Salience.AInsula (R) |
| Rostral prefrontal cortex (L) | Salience Network | (-32,45,27) | network.Salience.RPFC (L) |
| Rostral prefrontal cortex (R) | Salience Network | (32,46,27) | network.Salience.RPFC (R) |
| Supramarginal Gyrus (L) | Salience Network | (-60,-39,31) | network.Salience.SMG (L) |
| Supramarginal Gyrus (R) | Salience Network | (62,-35,32) | network.Salience.SMG (R) |
| Lateral prefrontal cortex (L) | Fronto Parietal Network | (-43,33,28) | network.FrontoParietal.LPFC (L) |
| Lateral prefrontal cortex (R) | Fronto Parietal Network | (41,38,30) | network.FrontoParietal.LPFC (R) |
| Posterior parietal cortex (L) | Fronto Parietal Network | (-46,-58,49) | network.FrontoParietal.PPC (L) |
| Posterior parietal cortex (R) | Fronto Parietal Network | (52,-52,45) | network.FrontoParietal.PPC (R) |

Table S3. Lists the specifics of the ROIs used in the RSFC network analysis.

##

## References

[1. T. Yarkoni, R. A. Poldrack, T. E. Nichols, D. C. Van Essen, T. D. Wager, Large-scale automated synthesis of human functional neuroimaging data. *Nat. Methods*. **8**, 665–670 (2011).](http://sciwheel.com/work/bibliography/237365)

[2. R. W. Cox, AFNI: what a long strange trip it’s been. *Neuroimage*. **62**, 743–747 (2012).](http://sciwheel.com/work/bibliography/4255219)
